# Standardized phantoms for quantitative cardiac MRI

**DOI:** 10.1186/1532-429X-17-S1-W36

**Published:** 2015-02-03

**Authors:** Katy Keenan, Karl F Stupic, Michael A Boss, Stephen E Russek

**Affiliations:** 1 NIST, Boulder, CO, USA

## Background

**Figure 1:**
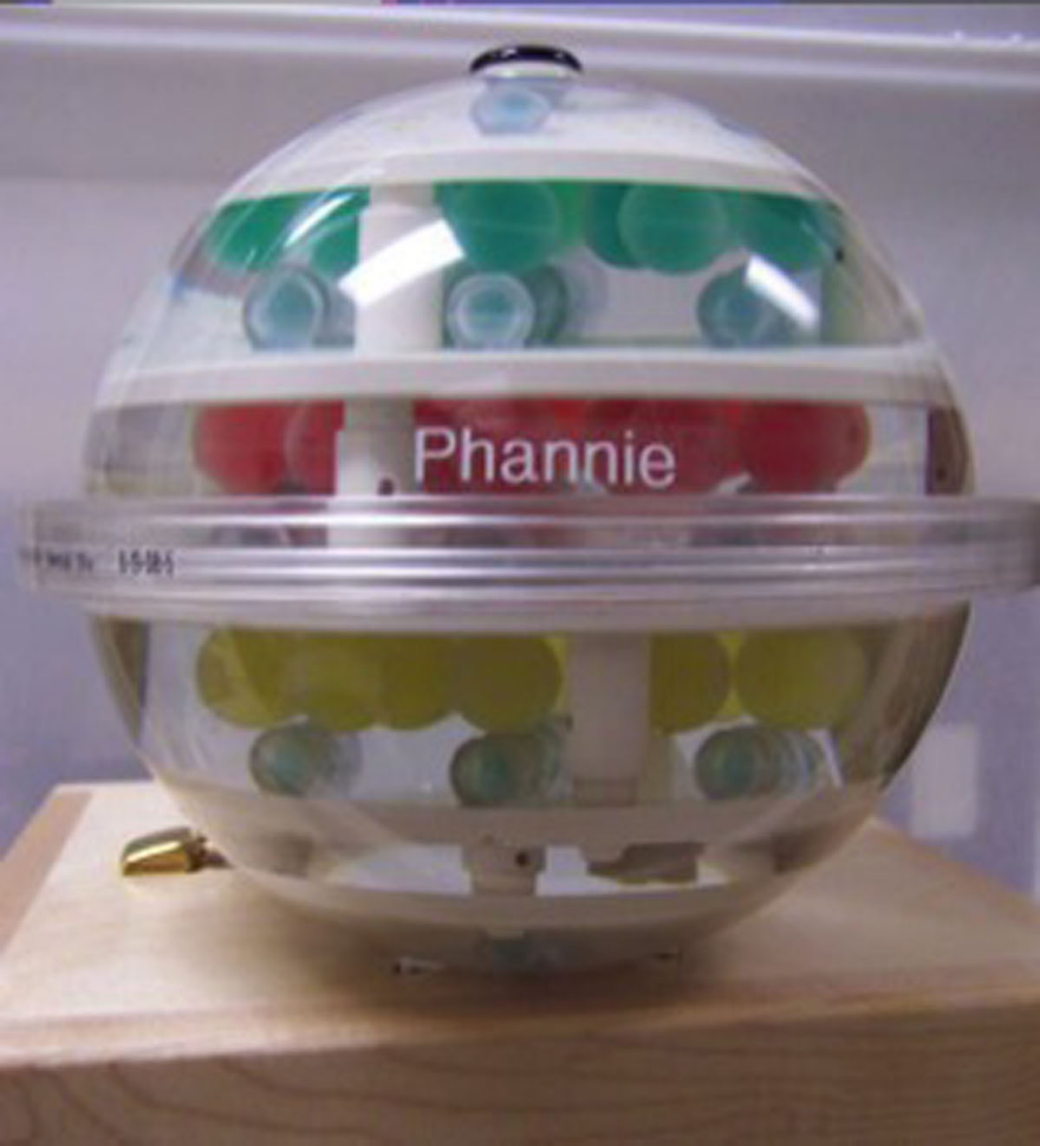
NIST/ISMRM system phantom to measure T1, T2, proton density and geometric distortions.

Quantitative MR relaxometry techniques are increasingly used in cardiac MR applications, e.g. MOLLI for high resolution T1 mapping. To use these techniques in the clinic, we must understand how to make comparable measurements across vendor systems and software versions and compare results across sites. A standardized phantom used for regular quality control is one approach. The Biomagnetic Imaging program at the National Institute for Standards and Technology (NIST) has created phantoms for measuring T1, T2, proton density in collaboration with ISMRM (Fig. 1) and diffusion in collaboration with RSNA-QIBA. In addition, a breast phantom including T1 and diffusion components is in development with UCSF. We propose a cardiac phantom focused on quantitative measurements of the myocardium pre- and post-contrast and the blood pool.

To test quantitative cardiac MR sequences, the phantom must mimic both the T1 and T2 relaxation properties in the same sample. The phantom must be stable, preferably for five years, and be reliably produced. Agarose gel is difficult to produce free of air bubbles and has a limited shelf life.

## Methods

We think the relaxation times can be mimicked using paramagnetic salts and metallic oxides in aqueous solution. By our calculations for 1.5 T, the post-contrast myocardium relaxation properties (T1 ~ 200 ms, T2 ~ 50 ms) can be mimicked using an aqueous solution of 8.32 mM NiCl_2_ and 0.36 mM MnCl_2_ or by 0.25 mM Feraheme solution. The pre-contrast myocardium and blood pool relaxation properties are more difficult. Based on experiments in our lab at 1.5 T, the pre-contrast myocardium relaxation properties (T1 ~ 1000 ms, T2 ~ 50 ms) can be mimicked using an aqueous solution of 0.1 mM FeRex. The blood pool relaxation properties (T1 ~ 1500 ms, T2 ~ 200 ms) can likely be mimicked using a combination of NiCl_2_ and Al_2_O_3_. Our work using Al_2_O_3_ is at a preliminary stage. The nano-iron solutions (Feraheme and FeRex) do have stability and susceptibility concerns that need to be explored further.

## Results

**Figure 2:**
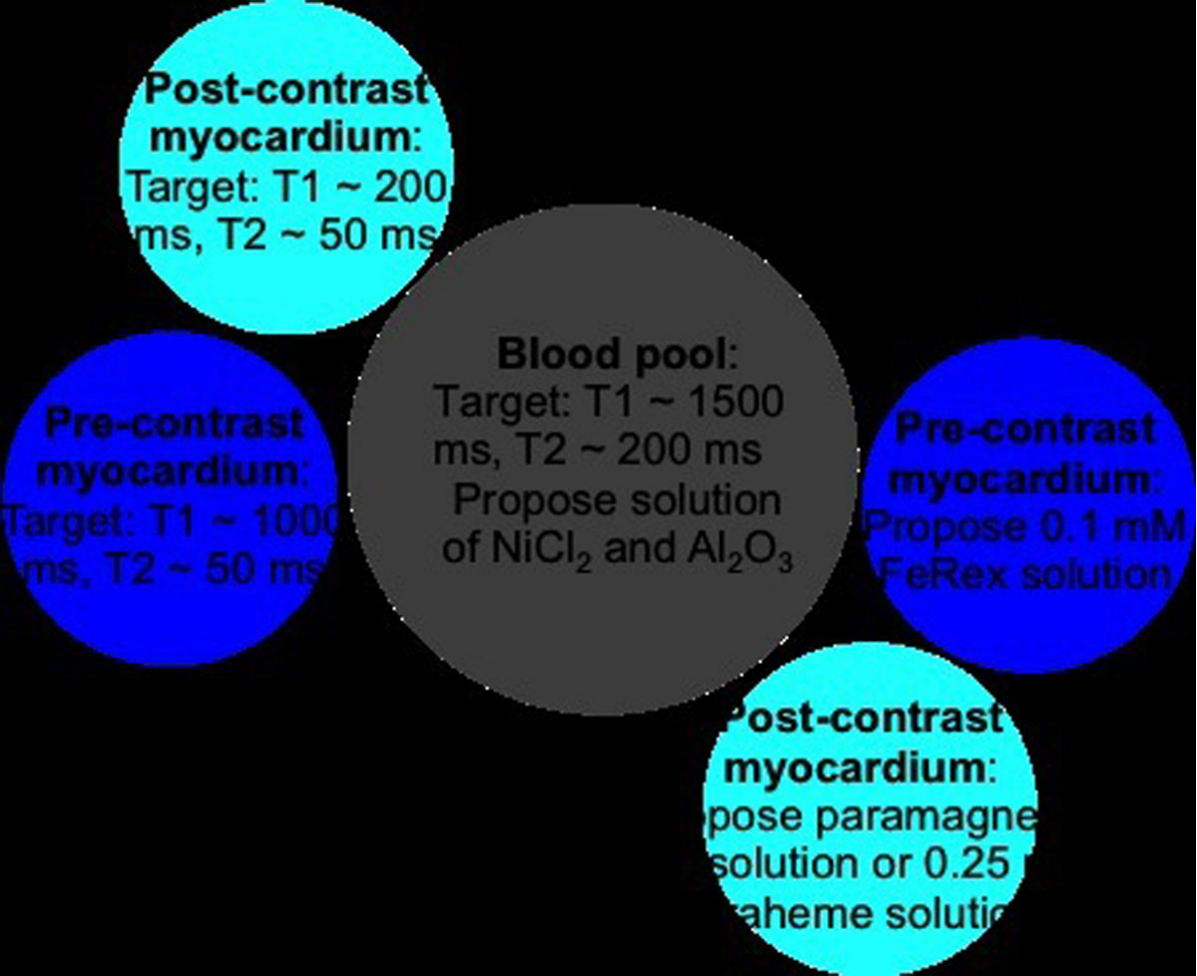
Proposed design for initial cardiac MR phantom.

We propose an initial design using readily available centrifuge tubes, suggesting a large tube (inner diameter 23 mm) for the blood pool and four smaller tubes (inner diameter 15 mm) for the pre- and post-contrast myocardium (Fig. 2). Future design could be an injection molded object with three compartments to better match the geometry of the left ventricle and healthy to diseased myocardium wall thickness (6 to 15 mm). In addition, a chest cavity material could surround future designs for proper loading of the coils.

## Conclusions

Future design could be an injection molded object with three compartments to better match the geometry of the left ventricle and healthy to diseased myocardium wall thickness (6 to 15 mm). In addition, a chest cavity material could surround future designs for proper loading of the coils.

## Funding

N/A.

